# Reforming a pre-existing biodiversity conservation scheme: Promoting climate co-benefits by a carbon payment

**DOI:** 10.1007/s13280-023-01833-4

**Published:** 2023-02-11

**Authors:** Johanna Kangas, Markku Ollikainen

**Affiliations:** Department of Economics and Management, University of Helsinki, P.O. Box 27, 00014 Helsinki, Finland

**Keywords:** Carbon payment, Carbon sequestration, Forest biodiversity, Payments for ecosystem services, PES

## Abstract

Protecting forests provides potential synergies for both biodiversity conservation and climate change mitigation. Payments for ecosystem services (PES) schemes are commonly used to promote biodiversity conservation in private forests, and including carbon as another target may be a cost-efficient way to promote both goals. We analyse a hypothetical reform on a forest biodiversity PES scheme by supplementing it with a carbon payment paid to landowners for also providing carbon benefits. With a site selection model, we examine how the proposed scheme could promote biodiversity and carbon values, and what level of the carbon payment would provide the highest synergy gains. We found that introducing the payment promotes both targets, but there is a temporal trade-off between selecting sites with high carbon storage or sites with good sequestration potential. The highest synergy gains are obtained in most cases by a second-best payment level of 10–20 € tCO_2_^−1^.

**Supplementary Information:**

The online version contains supplementary material available at 10.1007/s13280-023-01833-4.

## Introduction

The two major environmental crisis of our time, biodiversity loss and climate change, are deeply interconnected and require policy tools that address both as neither can be solved without resolving the other (Pörtner et al. 2021). Forest conservation provides synergy gains between biodiversity and climate goals. Conserving forests safeguards the existence of numerous forest-dwelling species currently under threat of extinction (IPBES 2018). In addition, it secures the huge stocks of carbon in forest biomass and soil; forests act as carbon sinks far beyond their harvesting age and continue to store carbon as old-growth forests (Luyssaert et al. 2008; Framstad et al. 2013). Although the use of wood provides greenhouse gas emission reductions by avoiding fossil emissions with substitution and stocks of carbon in harvested wood products, the negative effect of harvesting on forest carbon stocks exceeds the substitution benefits (Soimakallio et al. 2022).

Acknowledging the urgent time frame of climate change mitigation and halting the biodiversity crisis argues for quickly expanding the network of protected forest areas. This is also the objective of the 2030 Biodiversity Strategy of the European Union (European Commission 2020). The EU LULUCF regulation on land use, land-use change and forestry requires that no emissions are generated from the land-use sector during the period from 2021–2025 to 2026–2030 (European Commission 2021). The LULUCF sector plays a significant role in achieving the EU’s target of net-zero emissions by 2050.

Payments for ecosystem services (PES) schemes are a common voluntary policy tool applied to forest biodiversity conservation in private forest lands in many European countries (Hanley et al. 2012). PES mechanisms provide landowners incentives to manage their lands in more environmentally friendly manner, e.g. via protection or restoration (Wunder 2005; Engel et al. 2008). The application of current PES schemes to forest lands entails two weaknesses. First, current PES schemes typically target the delivery of a single ecosystem service, like biodiversity or carbon but not both at the same time (Wunder 2005; Jack et al. 2008). Omitting the promotion of synergies between multiple environmental goals has been challenged in the context of pollution control policy that provides an example of targeting multiple pollutants (Robertson et al. 2014).^1^ Second, by compensating mechanically for the conservation costs instead of paying for direct provision of ecosystem services, PES mechanisms seldom provide the best economic incentives to forest landowners.

In this paper, we examine how to tackle the above weaknesses in a pre-existing PES scheme targeting biodiversity that cannot be fully reformed. More specifically, we introduce carbon payments as another PES instrument to the scheme to promote the high synergies between biodiversity and carbon storages. Carbon payments represent a result- or incentive-based instrument in contrast to the practice-based compensation of conservation costs. Therefore, this reform would also make the initial PES scheme system more price sensitive and would likely invite more forest landowners to participate in the scheme. We examine which level of the carbon payment would provide the highest synergy between biodiversity and carbon sink. We assume that by accounting for the synergies between biodiversity and climate goals, the government is willing to allocate some climate mitigation funds to forest conservation (Deal et al. 2012; Matthies et al. 2016). Under these assumptions, the PES scheme becomes a hybrid model of practice- and result-based mechanisms. It is not optimal but second-best, as mechanism relying solely on incentives, such as tendering systems, would be superior. Despite this, we account here for the fact that changing a long-standing mechanism completely is difficult.

We apply our analysis to the Finnish PES scheme, METSO Forest Biodiversity Programme, which is the key policy tool in Finnish forest biodiversity conservation. It is based on landowners’ voluntary participation and offers both temporary and permanent conservation agreements. Thus, we examine how well adding carbon payments would work in this setting.^2^ Drawing on the actual data from the programme, we examine how the enrolment of forest sites and their composition change due to the introduced incentives in terms of ecological values, forest type and stand age. We search for the carbon payment level that would provide the highest synergy by letting the conservation budgets sizes and carbon payments to vary.

We employ a site selection model which is often used to solve conservation problems (e.g. Ando et al. 1998; Margules and Pressey 2000; Cabeza and Moilanen 2001; Polasky et al. 2001, 2005). Site selection modelling has been used to analyse the METSO programme in the previous literature. Juutinen et al. (2009) used the model to compare how a voluntary programme using a scoring procedure (a biodiversity index) succeeds in creating a representative conservation network compared to using the number of indicator species as a site selection criterion. They found that the scoring procedure is not as effective as using indicator species, but scoring was able to identify many of the sites hosting rare and threatened species. They pointed out that it is essential to attract large enough supply of sites so that the ecologically most valuable sites can be selected to the programme. Our analysis complements their work by examining the impact of increased supply from higher financial incentives. Our viewpoint is different in that we use only one biodiversity indicator as conservation criteria. We follow Kangas and Ollikainen (2022) and supplement the site selection criteria with a carbon index. Our analysis differs from theirs in that we employ price incentives instead of practice-based payments.

Given that forests provide at the same time carbon storage and carbon sink, the inclusion of carbon requires determination of how to weight the current storage of forest against its ability to sequester carbon. Therefore, we examine the temporal trade-off between storage and sink by selecting the sites and paying the carbon payment either on carbon storage or sequestration potential. Thus, we compare the options of conserving the best storages (protecting these sites from land use and logging) or selecting the sites where the carbon storages will grow the fastest during the next decades. In the former case, we account for a possibility that some sites with large carbon storages will be lost due to harvesting. As there is a clear difference in conservation costs between these options, there is also a choice between using more conservation budget on the expensive old-growth stands with high storages or conserving larger areas or spending less money on protecting the younger stands with potential sinks. Additionality is an important aspect to consider when promoting multiple environmental goals with one policy tool. We consider that additionality is satisfied at a policy level if supply responds to increased incentives.

Previous literature has employed ‘subsidise-and-tax’ forestry models to examine the impacts of a policy that uses subsidies to promote carbon sinks and taxes to penalize harvesting the carbon stock, as well as carbon stock policies that pay rents periodically based on the value of existing carbon stocks (van Kooten et al. 1995; Uusivuori and Laturi 2007; Lintunen et al. 2016). These full-scale forest-carbon policies encompass all commercial forest resources. In contrast, carbon offset programmes offer incentives to sequestrate carbon on a project level (UNFCCC 2011; Grafton et al. 2021). Our analysis combines carbon sequestration to biodiversity conservation and using a PES scheme resembles offsetting in that it incentivises the voluntary provision of targeted environmental gains. However, our PES scheme essentially differs from offsetting, as it is not set to compensate for any loss of biodiversity or GHG emissions elsewhere.

## Materials and methods

### Outline of the analysis

To analyse the implications to the conservation area network resulting from the reform in the PES scheme, we use a site selection model ("Site selection model" section). With the model, we can compare cost-effective alternatives for conservation under different targets and costs. The model compiles an optimal network of sites under the conservation budget by selecting stands so that each addition in biodiversity value (in the baseline) or combined biodiversity value and carbon (when the carbon payment is introduced) are as high as possible (Juutinen et al. 2008; Kangas and Ollikainen 2022).

Site selection in the METSO programme is based on habitat-type specific criteria that include structural features important for boreal forest biodiversity (old-growth stands, high amounts of dead wood, diverse stand structure including deciduous trees, aspens in particular) (Ministry of … 2015). We employ a composite biodiversity index (ELITE index, "Index values for biodiversity and carbon" section) that reflects these selection criteria.

The starting point of the analysis is the data: 100 forest stands protected permanently in the METSO programme. To simplify, we assume that the area of each site is one hectare. Based on the current status of the sites, we calculate biodiversity index values and the carbon storage. To enable the temporal comparisons, we also need to assess the potential future carbon sink they would provide. In both cases, we examine alternative levels of the carbon payment. We run forest simulations with a stand simulation model MOTTI^3^ over a 50-year time period. We then calculate the future carbon stock in 50 years based on the simulations.^4^ In addition, the payments to the future stocks are calculated.

The conservation budget constrains the choice. The budget is increased so that the conservation area does not decrease due to the additional cost from the carbon payment. The allocation of the conservation budget depends on the payments for the biodiversity and climate benefits ("The biodiversity and carbon payments" section). For biodiversity, we follow the current practice in METSO and assume that the payment compensates only for the value of the current tree stand. For climate benefits, we determine the payments based on current storage or sequestration potential, depending on the selection criteria.

A natural assumption is that the supply of sites increases as the overall payment to the landowner increases when the carbon payment is introduced. This makes conservation as an alternative to forestry more attractive, and it incentivizes landowners to offer sites that would not have been offered for the programme otherwise (Horne 2006; Juutinen and Ollikainen 2010; Lindhjem and Mitani 2012). Given the heterogeneity of forest sites in terms of biodiversity and carbon sequestration potential, paying for carbon in addition to biodiversity conservation helps a larger number of landowners to meet their opportunity costs and, thus, to increase the participation (von Hase and Cassin 2018). Several studies on voluntary participation in forest conservation programmes have shown that the level of the financial compensation is an important factor when forest owners decide whether to participate, or not (Boon et al. 2010; Mitani and Lindhjem 2015, 2021; Miljand et al. 2021). In the analysis, we consider price-elastic supply. Inelastic supply is examined in Appendix S2.

Suter et al.’s (2008) literature review of voluntary agri-environmental schemes found enrolment elasticities varying from 0.32 to 5; in their analysis, they calculated elasticities of 0.88 and 0.95 in the US Conservation Reserve Enhancement Program (CREP). We assume that the enrolment elasticity is 0.7, i.e. a 10% increase in the payment for landowners causes a 7% increase in the number of offered sites. We use a lower enrolment elasticity, because CREP is a more established program, and the landowners are better informed compared to our case. We introduce price elasticity in the analysis as follows. We use randomly selected subgroups of the 100 sites in the data and increase the group of offered sites by 7% for each 10% increase in the payment. With the highest carbon payment level, all 100 sites are considered in the selection of sites.

### Site selection model

We use a site selection model following Juutinen et al. (2008) and Kangas and Ollikainen (2022). We run the modelling with What is Best! spreadsheet optimization software (Lindo Systems 2000).

Consider $m$ different sites offered to be conserved in the METSO programme. The supply of sites, i.e. the landowner’s choice whether or not to participate in the programme, is determined by the relative size of the landowner’s opportunity costs and the biodiversity payment. A binary variable, ${x}_{j}$ ($j=1,...,m$), determines the status of a site; it obtains a value of 1 if the site is selected to be conserved and 0 otherwise. The biodiversity value of a site $j$ is denoted by ${e}_{j}$ and it ranges between 0 and 1. The conservation budget $(C)$ limits the number of sites that can be conserved. The biodiversity payment is denoted by ${c}_{j}$. In the baseline, the target is to maximize the sum of biodiversity values in the selected sites subject to the budget constraint:

1 $$ \mathop {\max }\limits_{{x_{j} }} \mathop \sum \limits_{j = 1}^{m} e_{j} x_{j}, $$

s.t.

2 $$ \mathop \sum \limits_{j = 1}^{m} c_{j} x_{j} \le C, $$

3 $$ x_{j} \in \left\{ {0,1} \right\}. $$

The objective function (1) sums the biodiversity values of the selected stands, constrained by the conservation budget (2), meaning that the sum of conservation costs cannot exceed the budget and by a technical requirement (3), meaning that the choice variables are binary, i.e. each site is either selected or not.

To modify the site selection, we add carbon in the objective function (4) and the cost from the carbon payment in the budget constraint (5). Furthermore, we consider the increased supply of sites. As the overall payment for the landowner increases, more landowners will choose to offer their land and consequently, the set of offered sites increases. We denote this extended set of supplied sites by $n$ ($n>m$). Furthermore, let ${d}_{j}$ denote carbon values, scaled between 0–1, and ${s}_{j}$ the cost accruing from the carbon payment:

4 $$ \mathop {\max }\limits_{{x_{j} }} \mathop \sum \limits_{j = 1}^{n} (e_{j} + d_{j} )x_{j}, $$

s.t.

5 $$ \mathop \sum \limits_{j = 1}^{n} (c_{j} x_{j} + s_{j} x_{j} ) \le C, $$

6 $$ x_{j} \in \left\{ {0,1} \right\}. $$

### Index values for biodiversity and carbon

We determine the numerical biodiversity value, ${e}_{j}$, of each site by using a so-called ELITE index, which is a habitat-based calculation method developed for estimating the state of Finnish habitats compared to their natural state (Kotiaho et al. 2016; Kangas et al. 2021). This index calculates ecological state using habitat-specific, ecologically most relevant structural components similar as in the METSO selection criteria: the amounts of dead wood, large trees, and depending on the forest type, broad-leaved trees (in fertile sites) or burnt area (in barren sites). Forest conservation prioritization in Finland has utilized similar biodiversity components (Moilanen et al. 2018; Forsius et al. 2021). As our data did not provide information on the number of large trees and burned area, we replaced large trees with stand age that correlates with tree size. The size of burned forest area in xeric and barren heath forests was ignored in the calculations due to data limitations. The biodiversity value is calculated for the current stand at the time of enrolment.^5^

The current state of the structural components is compared to a predefined reference state. Weights reflect the importance of each component to the overall state. The ELITE index values range from 0 to 1, where 1 is the reference state (natural or target state) and 0 implies that an ecosystem is completely degraded. The ELITE index calculates the biodiversity value as follows:

7 $$ e = \mathop \prod \limits_{n = 1}^{{N^{k} }} \left( {1 - L_{n}^{k} \left( {1 - \frac{{n_{curr} }}{{n_{ref} }}} \right)} \right), $$

where $e$ denotes the biodiversity value of the site. ${N}^{k}$ is the number of structural components for a habitat type $k$, ${L}_{n}^{k}$ is the weight for component $n$ and parameters ${n}_{curr}$ and ${n}_{ref}$ denote the current and the reference state of component $n$, respectively (Kotiaho et al. 2016). Parameter values for ${L}_{n}^{k}$ and ${n}_{ref}$ can be found in Table S1.1 in Appendix S1.

To determine a measure of the CO_2_ sequestered in the forest biomass, the amount of carbon dioxide, $D$, was calculated for the current stand and after the 50-year simulations as follows:

8 $$ D_{0} = v_{0} \left( {\left( {a + be^{ - 0,01t} } \right) \cdot 0,5 \cdot \frac{44}{{12}}} \right), $$

9 $$ D_{50} = v_{50} \left( {\left( {a + be^{ - 0,01t} } \right) \cdot 0,5 \cdot \frac{44}{{12}}} \right) - v_{0} \left( {\left( {a + be^{ - 0,01t} } \right) \cdot 0,5 \cdot \frac{44}{{12}}} \right). $$

Parameter ${v}_{0}$ stands for the stemwood volume of the current stand (current storage) and ${v}_{50}$ stands for the growth in stemwood volume after the 50-year simulation period (future sink). Stemwood volumes are converted into biomass (Mg m^−3^) using biomass expansion factors from Lehtonen et al. (2004) in the term $(a+b{e}^{-\mathrm{0,01}t}),$ where $t$ stands for stand age. Parameter values for $a$ and $b$ can be found in Table S1.2 in Appendix S1. Only the carbon stock of forest biomass (dead wood included) is calculated, excluding soil. The ratio 0.5 converts biomass to carbon, as 50% of the biomass is carbon (IPCC 2006), and the ratio of the molecular weights of carbon and carbon dioxide, 44/12, converts carbon to CO_2_. The figures for CO_2_ (${d}_{j}$ in Eq. (4)) are scaled between 0 and 1 by dividing each site’s CO_2_ value with the maximum value in the data.

### The biodiversity and carbon payments

The biodiversity payment (${c}_{j}$) is determined as the value of the current stand, i.e. the revenue from the current stand after an optimal rotation period:

10 $$ c_{j} = V\left( {t^{*} } \right)\left( {1 + r} \right)^{{ - t^{*} }} ,\;{\text{if}}\; t^{0} < t^{*}, $$

11 $$ c_{j} = V\left( {t^{0} } \right),\;{\text{if}}\;t^{0} > t^{*}, $$

where ${t}^{0}$ denotes the current stand age and ${t}^{*}$ the stand age in the end of the optimal rotation period. Parameter $r$ is the interest rate (3%) and $V$ is the value of the stand, either the current stand $(V\left({t}^{0}\right))$ or stand after optimal rotation period ($V\left({t}^{*}\right))$. Thus, given the focus of the paper, we follow the METSO biodiversity payment determination and do not account for the lost future revenue (i.e. Faustmannian bare land value), only the lost revenue from the current stand.

The carbon costs for each site (${s}_{j}$) are calculated by multiplying the carbon payment ($p$) by the amount of CO_2_ (storage or future stock after 50 years, ${D}_{j}$) and when paying for future sink, discounting to net present value as follows):

12 $$ s_{{0_{j} }} = p D_{{0_{j} }} , $$

13 $$ s_{{50_{j} }} = p D_{{50_{j} }} \left( {1 + r} \right)^{ - \tau } . $$

No unambiguous approach exists for pricing carbon and the literature focusing on climate benefits from forest uses different estimates (van Kooten et al. 1995; Pohjola and Valsta 2007; Asente and Armstrong 2012; Juutinen et al. 2018). Therefore, we used different estimates for the carbon payment. Estimates of the social cost of carbon, i.e. marginal economic damage caused by an additional tonne of carbon dioxide emissions, range from $15 to $33–106 tCO_2_^−1^ (Tol 2005, 2011). We search for a carbon payment that entails high synergy gains and used five alternative prices for the carbon payment ($$p$$): 10, 20, 30, 40 and 50 € tCO_2_^−1^. The lower prices work as a premium and the highest payment is equivalent to the carbon price in the EU ETS in 2021 (approximately 54 € tCO_2_^−1^) (EEX 2021). An interest rate ($$r$$) of 3% was used in discounting and the time period ($$\tau $$) was 50 years.

### Data

The data consist of 100 sites permanently protected in the METSO program. The study sites are located in North Karelia in eastern Finland and in Southwest Finland and Satakunta in western Finland. Parks & Wildlife Finland, which manages state-owned nature reserves in Finland (SAKTI 2019), has collected the data. The data have been gathered on field assessments and they include information on the forest-site type, tree species, the volume of broad-leaved trees, stand age and the amount of decaying wood. The data cover all Finnish forest site types (classified based on fertility, see e.g., Tonteri et al. 1990),^6^ different age classes (6–230 years) and different tree species compositions. Table S1.3 in Appendix S1 presents the basic information of each stand in the data and Table S1.4 gives the biodiversity and CO_2_ values as well as biodiversity and carbon payments calculated for each site.

## Results

We start by creating a baseline for site selection in the METSO programme and introduce the carbon payment as a reform of the mechanism. We employ a fixed conservation budget and carbon payment (20 € tCO_2_^−1^) that is levied either on the current carbon storage or on the sequestration potential of the site. We examine in detail the synergies and trade-offs between biodiversity and carbon values for this selection. In the next section, we explore, which size of the carbon payment would provide the highest synergy gains by varying conservation budgets and the level of carbon payments each budget.

### Introducing the carbon payment: the composition of the conservation network after the reform

Sites under the current METSO baseline are chosen cost efficiently according to their biodiversity values and conservation costs as the biodiversity payment. The first column in Table 1 shows the results. The next two columns present the results when the carbon index is added to the site selection targets and the carbon payment (20 € tCO_2_^−1^) is included as a cost in the conservation budget. Supply of sites is price elastic, i.e. the supply responds to the increased financial incentives. The level of the budget was determined by calculating the cost of a carbon storage payment or potential sequestration payment in the baseline and adding + 10%. The supply of good-quality sites increases as a result of the carbon payment, and therefore, conservation budget must be increased by a small margin of 10%.^7^ Recall also that the elasticity of the supply was assumed to be 7%.

**Table 1:** Results in the baseline and when the carbon payment is introduced (under price-elastic supply, carbon payment 20 € tCO_2_^−1^)

	Baseline	Current carbon storage	Carbon sequestration potential
Conservation costs, total, €	299 710	560 750	418 960
Average BD value × area	0.31 × 57 = 17.6	0.31 × 61 = 19.1	0.30 × 63 = 18.7
Storage, tCO_2_	10 470	11 800	10 860
Potential sink, tCO_2_	17 720	17 940	20 650
Combined index value (incl. storage)	35.1	38.8	36.9
Combined index value (incl. sequestration potential)	44.4	46.3	50.0
Average stand age, years	80	82	72

Biodiversity (BD) values are expressed as average index values of the selected sites and total sums (average value × area). Carbon values are expressed as tonnes of CO_2_ and combined index values are calculated as the sum of biodiversity and carbon index values, including either carbon storage or potential sink. For the baseline, we report ex-post carbon storage and carbon sequestration potential.

Table 1 shows that the budget increase due to the reform leads to larger conserved land areas. Compared to the baseline, introducing carbon payment increases biodiversity values in total, as the conserved area increases but the average values do not change (current storage) or decrease only slightly (sequestration potential). The carbon storage selection invited older stands in the programme than the baseline selection, whereas the potential sink selection leads to younger stands. The sizes of the current carbon storage and the potential sink increase from the baseline after the reform. The combined index values increase clearly in both options as a result of the area increase and increases in the carbon index values. Not surprisingly, paying and selecting sites based on current storage leads to higher storage than considering potential sequestration and vice versa. When sites are selected according to their sequestration potential, some sites with high carbon storages are left out of the conservation network. Assuming that these sites will be in commercial use suggests that 13% more current carbon storage is lost in harvests when sites are selected based on their sequestration potential. Appendix S2 presents results for a carbon payment of 10, 30, 40 and 50 € tCO_2_^−1^, for inelastic supply and when potential biodiversity values are considered instead of current ones.^8^

**Fig. 1:**
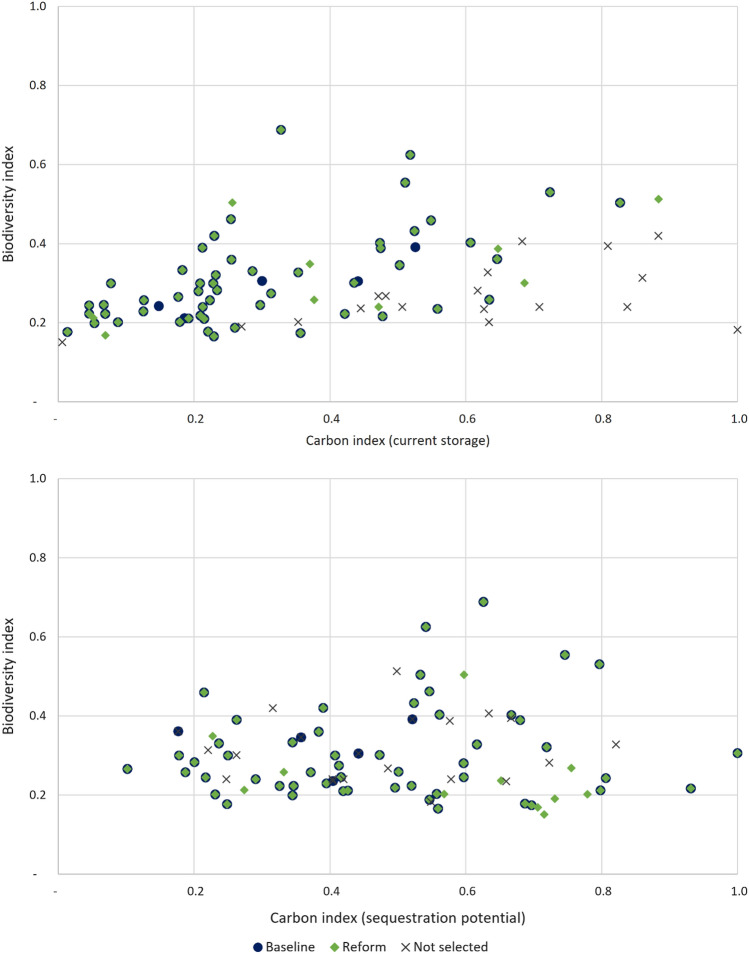
Biodiversity (y-axis) and carbon (x-axis) indices of selected sites, selection based on current carbon storage (above) and carbon sequestration potential (below)

Figure 1 illustrates biodiversity and carbon values of the selected sites in the baseline and with the carbon payment. The synergy gains increase when moving up and on the right in the figure. In the upper panel (payment for the storage), many of the best carbon storages are not selected in the baseline or after the reform. This shows the impact of the higher cost of the carbon payment: some of the best storages do not become selected due to their high cost. When the sites are selected based on carbon sequestration, more new sites selected have a good carbon index value ($>0.5$). In addition, the sites not selected are mainly located on the left-hand side in the figure. Figure 1 also shows that the difference between the baseline and the reform is quite modest and mainly the same sites become conserved.

The cost from the carbon payment paid based on the storage is approximately threefold compared to the payment from the future sink when the payment is 20 € tCO_2_^−1^. The costs of the scheme increase on average approximately 3900 € ha^−1^ when paying for the storage and 1500 € ha^−1^ when paying for the potential sink. This would require an increase in the conservation budget of at least 75% (storage) or 26% (future sink). If we set these figures in proportion of the current budget of the METSO programme, the carbon payment would require a budget increase from 240 M€ to 420 M€ (storage) or to 304 M€ (future sink) in 2026–2030.

**Fig. 2:**
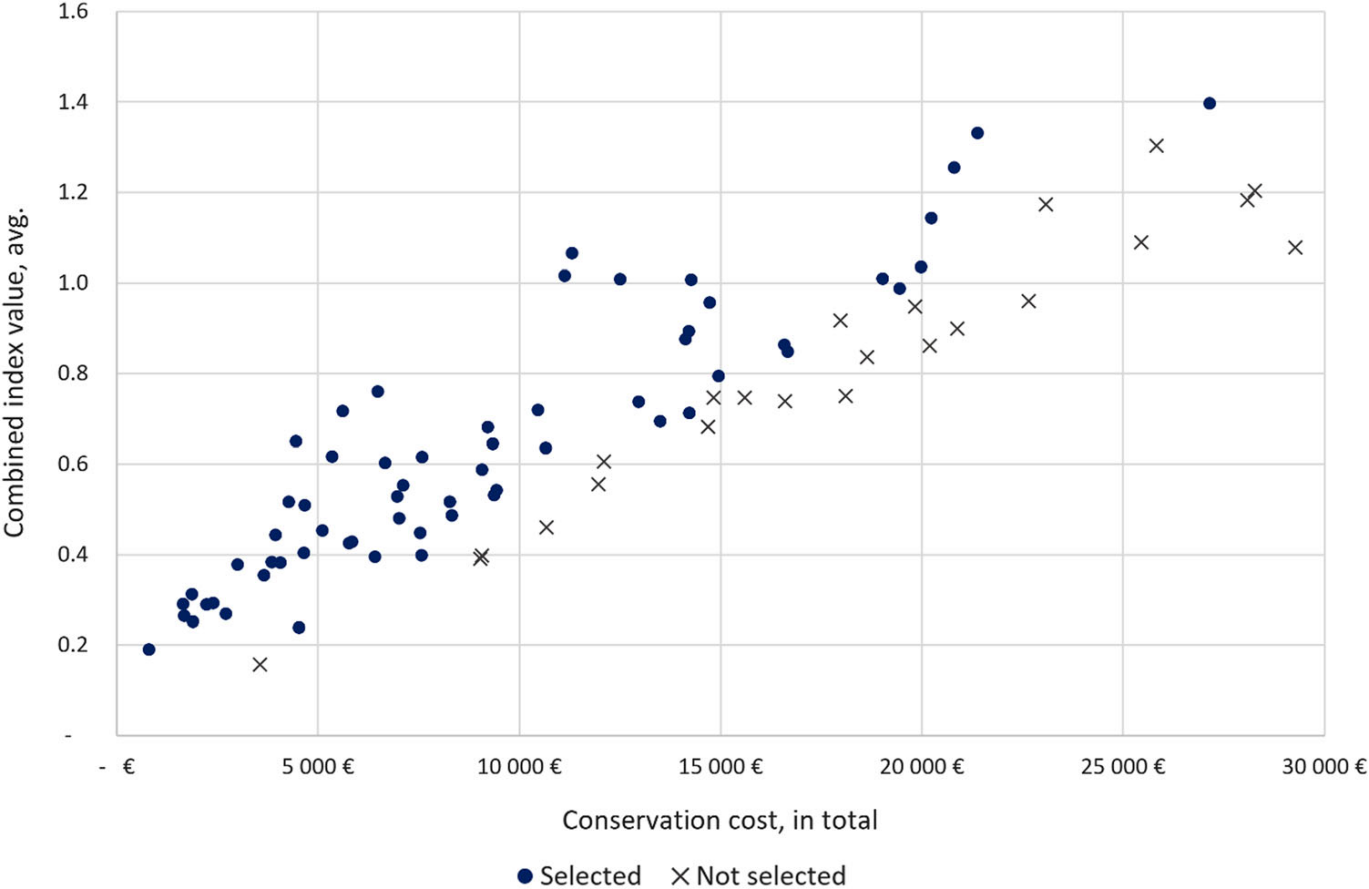
The total conservation cost (including the biodiversity and carbon payment) and combined index value per hectare of the selected sites (20 € tCO_2_.^−1^, current storage)

Figure 2 depicts the combined index values of the selected sites (incl. current carbon storage) on the y-axis and total cost of conservation (the biodiversity and carbon payment) on the x-axis. The figure shows that the site selection conserves the best sites on any given level of costs but the further right in the figure, the less sites become selected, reflecting the impact of the high cost of conserving these sites. For example, among the sites that cost 20 000–25 000 € ha^−1^, three sites is conserved and 25 000–30 000 € ha^−1^, only the best site is conserved.

### Optimal level for the carbon payment: examining various payments and conservation budgets

We ran the site selections for five alternative payment levels (10–50 € tCO_2_^−1^) under differing conservation budgets (300 000–900 000 €) to see which payment level leads to the highest synergy gains. Tables 2 and 3 present the results for average biodiversity index and carbon index values in the selected sites and Table 4 shows the combined index values on average. Grey colour indicates the highest average value for each budget. Table 2 shows the results when the carbon payment is paid based on the current carbon storage and Table 3 for the carbon sequestration potential.

**Table 2:** Results for the carbon payment based on the carbon storage

Budget, €	Average biodiversity index values					Average carbon index values				
	*10 €*	*20 €*	*30 €*	*40 €*	*50 €*	*10 €*	*20 €*	*30 €*	*40 €*	*50 €*
300 000	0.30	0.31	0.30	0.31	0.30	0.29	0.26	0.23	0.22	0.19
400 000	0.31	0.31	0.32	0.31	0.30	0.33	0.28	0.27	0.23	0.20
500 000	0.31	0.31	0.31	0.30	0.31	0.35	0.30	0.28	0.25	0.22
600 000	0.30	0.31	0.31	0.31	0.31	0.38	0.33	0.30	0.28	0.25
700 000	0.30	0.31	0.31	0.31	0.30	0.38	0.35	0.32	0.29	0.27
800 000	0.30	0.31	0.31	0.31	0.30	0.38	0.36	0.34	0.30	0.27
900 000	0.30	0.30	0.31	0.31	0.31	0.38	0.38	0.35	0.31	0.29

**Table 3:** Results for the carbon payment based on the carbon sequestration potential

Budget, €	Average biodiversity index values					Average carbon index values				
	*10 €*	*20 €*	*30 €*	*40 €*	*50 €*	*10 €*	*20 €*	*30 €*	*40 €*	*50 €*
300 000	0.29	0.29	0.28	0.28	0.28	0.48	0.48	0.49	0.48	0.46
400 000	0.30	0.30	0.29	0.28	0.28	0.50	0.49	0.48	0.49	0.48
500 000	0.30	0.30	0.29	0.29	0.29	0.50	0.49	0.48	0.48	0.48
600 000	0.30	0.30	0.30	0.29	0.29	0.49	0.50	0.49	0.49	0.49
700 000	0.30	0.30	0.30	0.29	0.30	0.49	0.49	0.50	0.49	0.49
800 000	0.30	0.30	0.30	0.30	0.30	0.49	0.49	0.49	0.50	0.50
900 000	0.30	0.30	0.30	0.30	0.30	0.49	0.49	0.49	0.50	0.50

**Table 4:** Results for the combined index values in both options

Budget, €	Current carbon storage payment					Carbon sequestration potential payment				
	*10 €*	*20 €*	*30 €*	*40 €*	*50 €*	*10 €*	*20 €*	*30 €*	*40 €*	*50 €*
300 000	0.59	0.58	0.53	0.53	0.48	0.77	0.77	0.77	0.76	0.74
400 000	0.64	0.59	0.59	0.55	0.50	0.80	0.78	0.77	0.77	0.76
500 000	0.66	0.62	0.59	0.55	0.53	0.80	0.79	0.77	0.77	0.77
600 000	0.68	0.65	0.61	0.59	0.56	0.79	0.81	0.79	0.79	0.79
700 000	0.68	0.66	0.63	0.60	0.57	0.79	0.79	0.80	0.79	0.79
800 000	0.68	0.67	0.65	0.61	0.57	0.79	0.79	0.79	0.79	0.80
900 000	0.68	0.68	0.65	0.62	0.60	0.79	0.79	0.79	0.80	0.80

Comparing the average carbon index values shows that when paying for current storage (Table 2), the 20 € payment almost always leads to the highest biodiversity index values. For higher budgets, a 30 € carbon payment occasionally leads to better or equal outcomes but the differences in the biodiversity values are altogether very small. The 10 € payment always leads to the highest carbon values. This finding results from the fact that paying for carbon storage is expensive, and conservation budget becomes quickly exhausted. Paying for potential carbon sequestration (Table 3) is cheaper, and therefore, the conservation budget leads to a selection with more sites. Now 20 € payment for carbon sequestration leads to the highest BD values with two exceptions. There is more variation in the carbon values. The highest values are obtained with 10 € payment for lower conservation budgets but the values increase slightly with large budgets and carbon payments. This feature results because we employ the same conservation budgets for both cases—the budget becomes de facto the laxer at higher budget levels distorting the comparison. In any case, a 20 € payment seems to work best in regard to biodiversity values but with carbon values, it depends more on the budget.

In Table 4, we assess the combined index values. It shows that carbon payment 10 € for carbon storage leads to the highest combined values apart from the highest budget. Thus, taken together Tables 2 and 4 suggest that the carbon payment of 10 € tCO_2_^−1^ for carbon storage maximizes the synergy between biodiversity and carbon. If the payment is based on carbon sequestration potential, the best combined index slides towards higher payments with the higher conservation budgets because the budget becomes laxer. Interestingly, for lower conservation budgets, the optimal value tilts to 10 € from 20 €. Thus, the results suggest that the carbon payment is really a second best, not reflecting entirely the social benefit from carbon sequestration. This finding is in line with the classical theory of second best (Lipsey and Lancaster 1956).

**Fig. 3:**
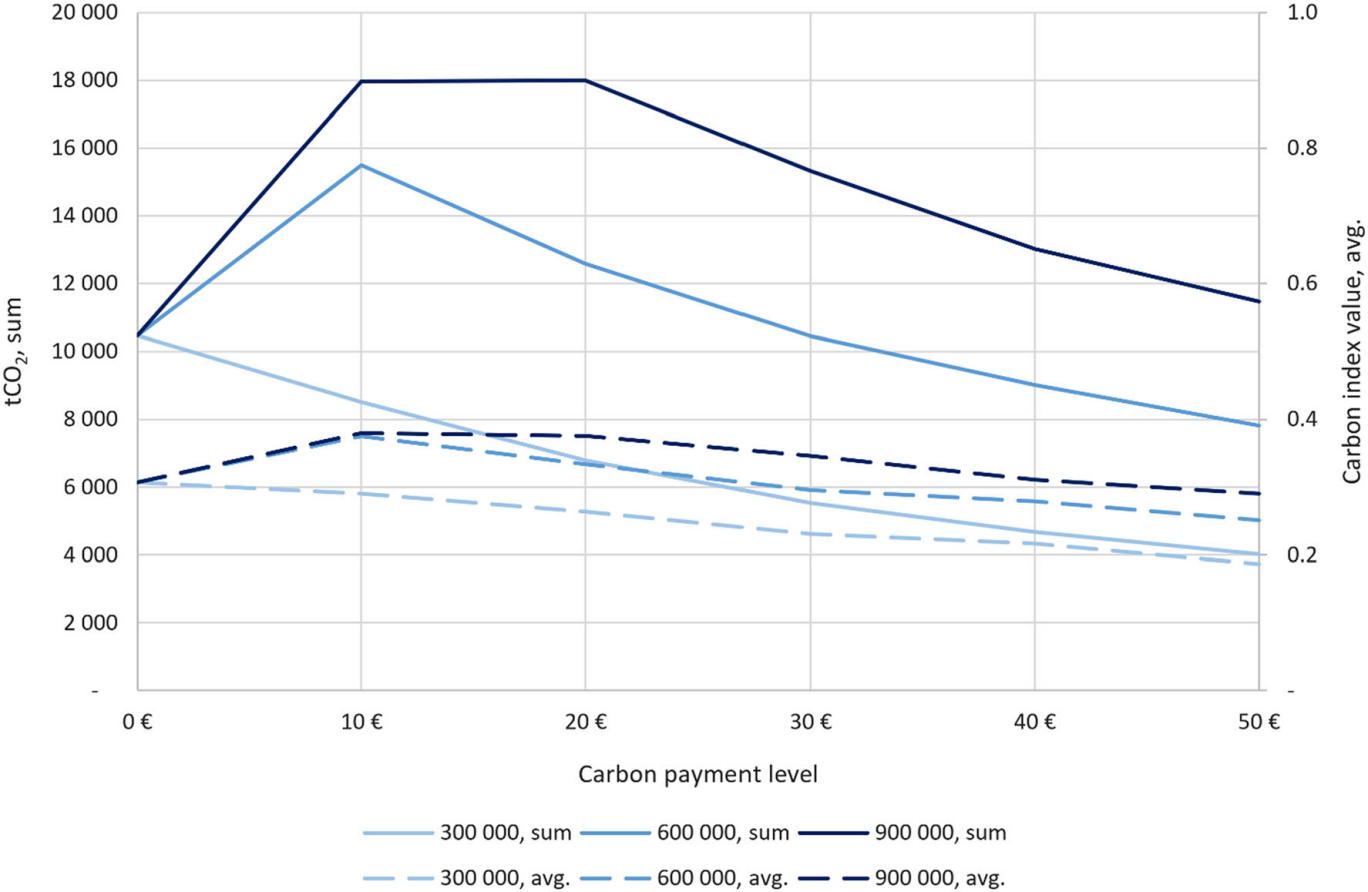
Results for carbon values (current carbon storage): tCO_2_ sums (y-axis on the left) and carbon index value averages (y-axis on the right) and the carbon payment levels on the x-axis

**Fig. 4:**
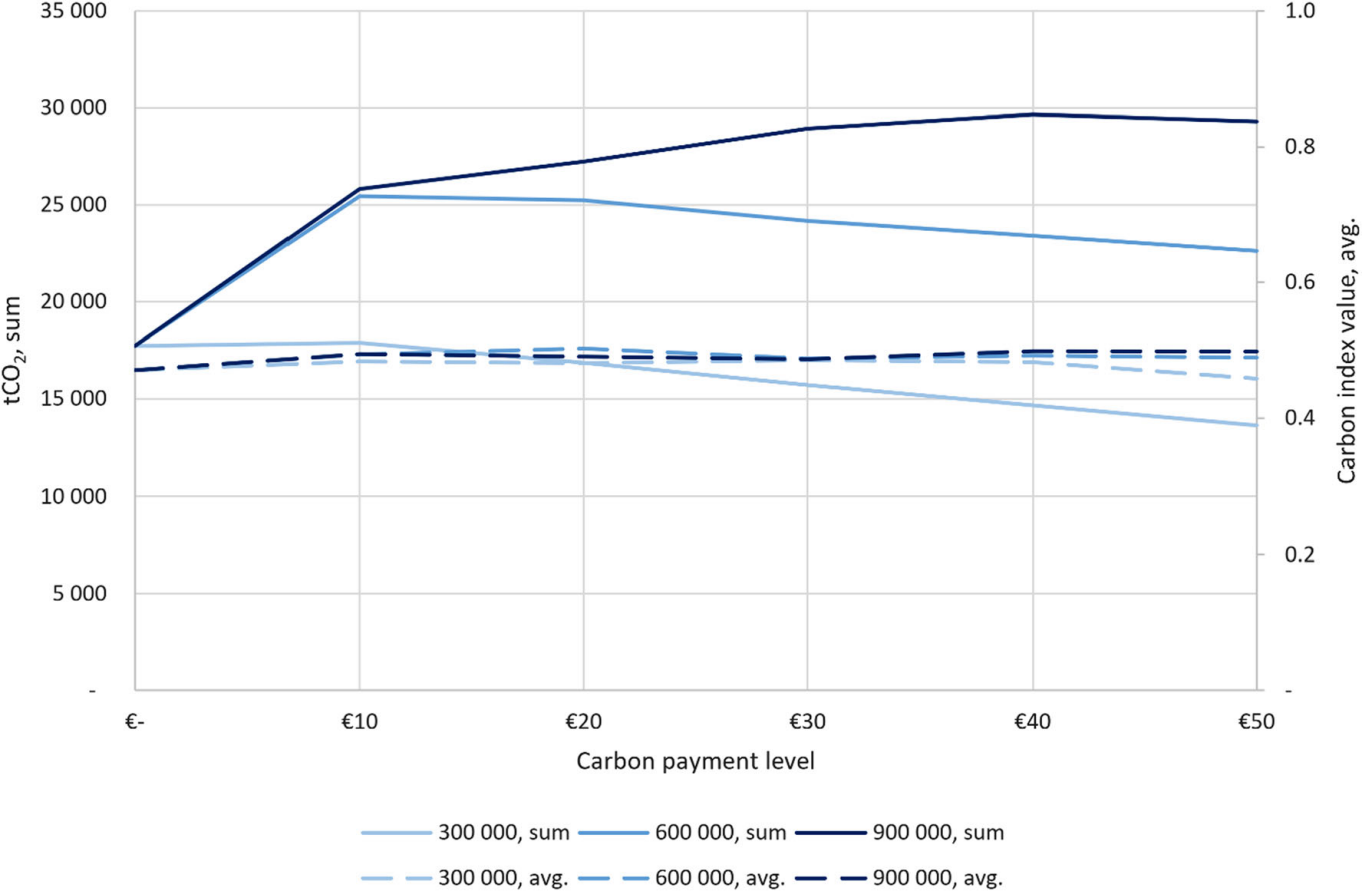
Results for carbon values (carbon sequestration potential): tCO_2_ sums (y-axis on the left) and carbon index value averages (y-axis on the right) and the carbon payment levels on the x-axis

To get further insight in the synergy maximizing values, we illustrate the averages and sums of index values from Tables 2 and 3 in Fig. 3 and 4. The results for carbon values, both sum (y-axis on the left) and average (y-axis on the right) and for three conservation budgets. The figures show that with the current storage payment: regardless the size of the budget, the average values are the highest with the 10 € payment. The sums are the highest with the 10 € payment as well, apart from the highest budget where the sum peaks at 20 € payment. Both the averages and sums decrease when the payment increases regardless of the budget because of the high cost from the carbon storage payment. Figure 4 differs from Fig. 3 because with the payment for potential sequestration, the peak in carbon average values depends directly on the conservation budget. The averages and sums do not decrease when the payment is increased as clearly as in Fig. 3 but instead, they increase under the highest budget.

**Fig. 5:**
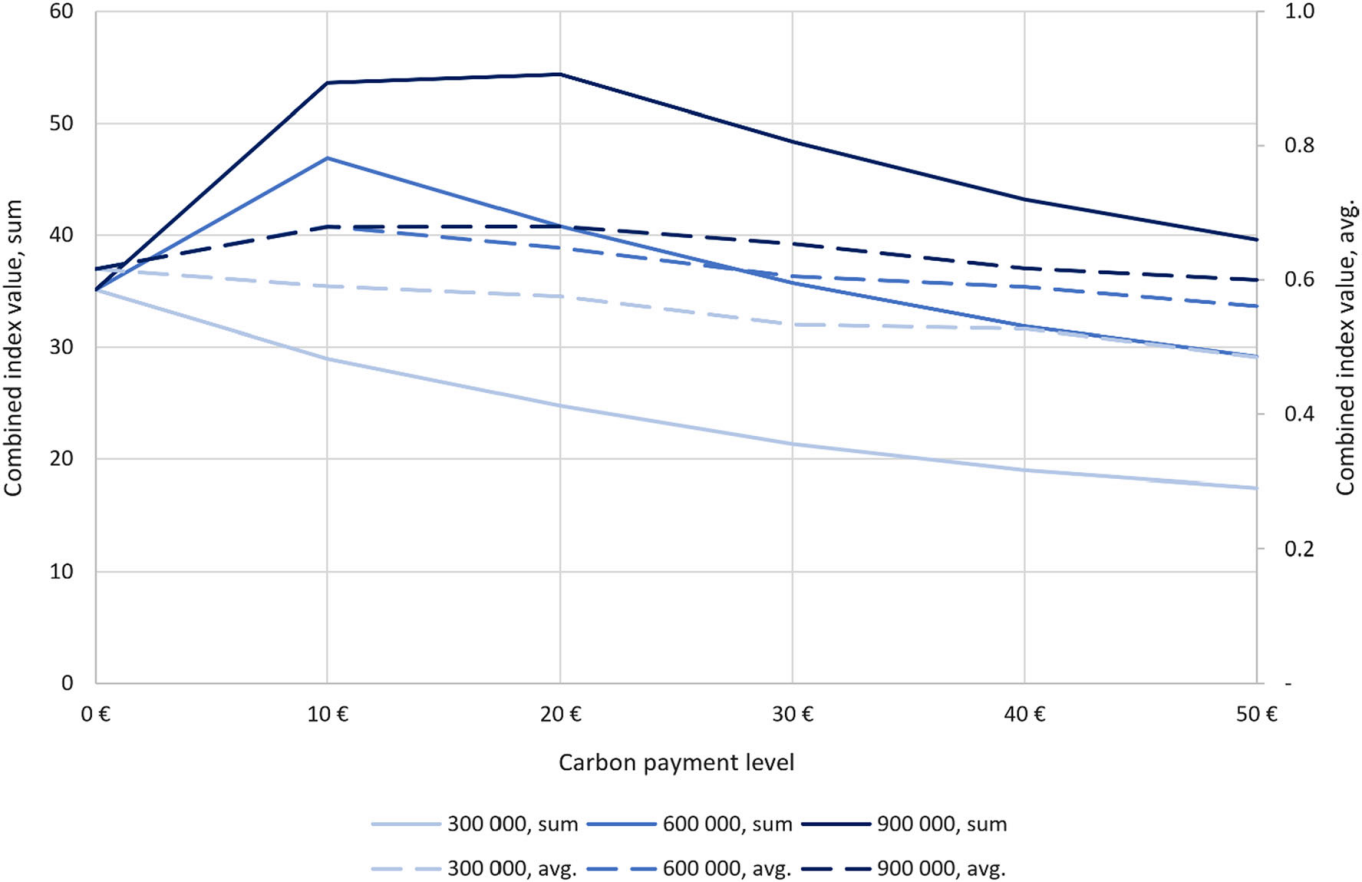
Results for combined index values (current carbon storage): sums on the y-axis on the left and averages on the right

Figure 5 presents the results for combined index values in the case of current storage selection. Sums are depicted on the y-axis on the left and averages on the right. The figure shows that although the peaks in biodiversity values vary (Table 2), they do not change significantly after the reform and thus, the shape of the curves are much the same as in Fig. 3.

## Discussion

In this study, we used the Finnish METSO programme as a case study for a reform where a carbon payment is added to a biodiversity conservation PES to achieve synergy gains for both biodiversity and climate goals. We compared two options for reform: selecting the sites drawing on current biodiversity values and targeting the carbon payment either on current carbon storage or carbon sequestration potential. We examined what size of carbon payment would provide the highest synergy gains between the biodiversity and climate goals.

Our results show that introducing a carbon payment to a biodiversity conservation programme with an equivalent increase in conservation budget increases supply of sites and promotes synergy between biodiversity and carbon targets. Both the levels of the carbon payment and the conservation budget play an important role. A higher budget leads to a larger land area being conserved which also increases synergy gains in total but high carbon payment levels increase the cost of conserving the sites with the highest carbon values. We also demonstrated that carbon target—conserving best carbon storages or sites with highest carbon sequestration potential in the coming decades—matters a lot for the nature of the conservation area network. The risk of losing carbon hotspots by harvesting emphasizes the choice of carbon stock over potential sequestration and so does the urgent time frame of emission reductions.

Our main result is that a second-best carbon payment (a “premium”), compensating only for a part of the climate benefits, brings the highest synergy gains for both targets. When paying for carbon storage, a payment of 10 € obtains the highest synergy gains in most of the cases. When paying for sequestration potential, the highest synergy gains depend more on the conservation budget but when the budget is scarce, 10 or 20 € premiums lead to the highest synergy gains. These findings are in line with the theory of second best, which indicates that if one variable is fixed at a non-optimal level, the second-best solution involves changing other variables away from the values that would otherwise be optimal. A second-best result-based payment could increase considerably the benefits from current biodiversity conservation programmes. A hybrid of practice- and result-based payments leads to a better outcome than a solely practice-based scheme.

Our results are in line with previous literature. Mäntymaa et al. (2009) found that higher payments correlated with higher ecological values when trading in nature values was piloted in Finland with the METSO program. Kumela and Koskela (2006) found that in the same pilot, landowners would have offered sites with higher stand density and fertility (i.e. higher carbon storage and likely higher biodiversity values) if a higher conservation payment was offered. Finally, our analysis of the temporal trade-off between carbon targets confirms the findings of Kangas and Ollikainen (2022).

The carbon payment levels employed in our analysis can be compared to abatement costs per tCO_2_ or marginal abatement costs in other sectors. The carbon price in the EU ETS was on average approximately 54 € tCO_2_^−1^ in 2021 and 80 € tCO_2_^−1^ in 2022 (EEX 2021; EEX 2022). Abatement costs in the agricultural sector are estimated to vary from 20 to over 100 € tCO_2_^−1^-eq in 2023–2040 (Koljonen et al. 2021). In Finland, over all sectors, the marginal abatement cost is estimated to increase to approximately 50 € tCO_2_^−1^ in 2025 and 95–120 € tCO_2_^−1^ in 2030, assuming that the climate neutrality goal is achieved in 2035 (Koljonen et al. 2021). As our results suggest that 10 or 20 € carbon premiums lead to the highest synergy gains, this policy tool would offer a cost-effective option to promote GHG sinks in comparison to other measures to reduce emissions.

There is uncertainty related to the stand simulations which may lead to overestimations of the growth of the stand and, consequently, the carbon sequestration potential. The limited size of the data (100 sites) decreases the generalizability of the results but was sufficient to allow the comparisons of multiple budgets and payment levels and to examine the impact of increasing supply. The site selection does not fully correspond to the METSO programme as the sites are selected based on case-by-case examination of the selection criteria. However, conservation prioritization using partly the same structural characteristics important for biodiversity than the index used here has been conducted to support the site selection in the METSO programme (Moilanen et al. 2018).

The issue of additionality is related to promoting multiple environmental goals under one policy tool. The question in our case is, whether the carbon payment increases the carbon storage or potential sink of the conservation network against a baseline (paying only for biodiversity) or not. We conceive that additionality is satisfied in the group of offered sites because when the overall payment increases, conservation as an alternative to forestry becomes more attractive. This will incentivize landowners to offer sites that would not have been offered for the programme otherwise. Explicitly valuing and paying for carbon in addition to the biodiversity helps landowners to meet opportunity costs and thus, to increase the participation and the area of land offered (von Hase and Cassin 2018). This is an important factor, because the efficiency of such voluntary schemes largely depends on their ability to offer a competitive alternative to commercial use.

## Conclusions

A PES scheme promoting multiple goals provides a new tool to achieve the goals of the EU Biodiversity Strategy and the requirements of the LULUCF regulation. Our main findings can be condensed as follows. First, supplementing a pre-existing practice-based PES scheme with a result-based instrument increases the efficiency of the whole mechanism by improving the landowners’ incentives to participate in the scheme. Second, a combination of biodiversity and carbon payments that maximizes synergy between the two goals entails using a second-best carbon payment, that is, setting the payment below the first-best price of carbon. Third, the carbon payment should primarily be paid for carbon storage instead of carbon sink. The urgent time frame of climate change mitigation argues for paying for the current storage. In addition, biodiversity values and stand age correlate more with the carbon stock than the potential sink.

Drawing on the main findings, this analysis shows that existing practice-based PES schemes can be improved if a fundamental revision of the programme towards a result-based scheme is not feasible. Introducing a hybrid of practice- and result-based instruments increases the efficiency of solely practice-based tools. In the absence of a separate policy tool for carbon storages, an incentive-based instrument such as analysed here is the best tool for securing carbon storage in privately owned forests. However, if there is a need to strengthen the carbon sink in commercial forests, a separate instrument would be needed. Introducing an additional incentive-based instrument in forest conservation may likely promote voluntary conservation for a wider group of forest owners. As conservation would become a relevant option for more landowners, the possibilities of achieving the biodiversity goals in forests with voluntary mechanisms would likely increase.

### Supplementary Information

Below is the link to the electronic supplementary material.

Supplementary file1 (PDF 533 kb)

## Footnotes

1 Targeting multiple goals from a single project is called stacking, which means that landowners are allowed to collect revenues for providing ecosystem services or abatement of several pollutants from the same activity or land area and to then sell the stacked credits individually to different buyers (Robertson et al. 2014). In contrast, bundling refers to selling different co-benefits as a single package to the same buyer. As many environmental problems are linked, developing market-based multi-pollutant approaches increases a policy’s efficiency (Robertson et al. 2014; Reeling et al. 2018).

2 Bundling in PES can be implemented in the form of a premium paid to landowners for offering co-benefits in addition to the main ecosystem service in focus (von Hase and Cassin 2018). This can help landowners to meet their opportunity costs and may increase the number of participants and area of land offered to the schemes, may reduce the high transaction costs of establishing individual markets for carbon and biodiversity conservation and limit the possible trade-offs between these goals (Deal et al. 2012; von Hase and Cassin 2018).

3 The MOTTI software has been developed in the Natural Resources Institute Finland. It predicts the growth of forests based on key results of forest growth and yield research in Finland (Hynynen et al. 2005).

4 We also calculate potential biodiversity values based on the 50-year stand simulations. The site selection results considering potential biodiversity values are represented in Appendix S2.

5 The biodiversity value in 50 years, calculated based on the simulations, is used in Appendix S2.

6 Forest-site types are categorized in the Finnish system into a hierarchy that reflects increasing capacity of the sites to produce stemwood, from dry and barren sites to moist and fertile ones (Tonteri et al. 1990).

7 Without the 10% addition to the budget, the synergy gains still exist, but they are more modest as the budget is not sufficient to select the more valuable sites.

8 The case of inelastic supply shows that the biodiversity and carbon values are higher than in the baseline but lower than in with elastic supply, i.e. as the set of offered sites remains constant, the synergy gains from the carbon payment are more modest but they still exist.
