# Supplementary material for: Reforming a pre-existing biodiversity conservation scheme: Promoting climate co-benefits by a carbon payment
Source: Ambio. 2023 Feb 11;52(11):1847–60. doi: 10.1007/s13280-023-01833-4 (PMC10562355; doi:10.1007/s13280-023-01833-4)
Supplement: Supplementary file 1 — Supplementary file1 (PDF 533 kb) [file 13280_2023_1833_MOESM1_ESM.pdf]

Ambio

Supplementary Information

This supplementary information has not been peer reviewed.

Title: **Reforming a pre-existing biodiversity conservation scheme: promoting climate co-benefits by a carbon premium**

Appendix S1

Table S1.1 Parameter values for calculating the ELITE index

	Structural components	*Reference values*	*Weights*
Herb-rich forests	Dead wood, m^3	100	0.6
	Broad-leaved trees, m^3	100	0.4
	Age*, years	100	0.4
Heathland forests: OMT, MT, VT	Dead wood, m^3	80	0.4
	Broad-leaved trees, m^3	50	0.6
	Age*, years	120	0.4
Heathland forests: CT, CIT	Dead wood, m^3	40	0.4
	Age*, years	140	0.4

*Replaces the component of large trees

Table S1.2 Parameter values for the biomass expansion factors (Lehtonen et al. 2004)

	a	*b*
Scots pine	0.7018	0.0058
Norway spruce	0.7406	0.1494
Broad-leaved trees	0.5616	-0.0179

Table S1.3 Data: forest site types, initial stand age, dominating tree species and components of the ELITE index. Abbreviations: OMaT = herb-rich forest, OMT = herb-rich heath forest, MT = mesic heath forest, VT = sub-xeric heath forest, CT = xeric heath forest, CIT = barren heath forest.

Site number	*Forest type*	*Dominating tree species*	*Average stand age, years*	*Dead wood, m^3*	*Broad-leaved trees, m^3*
1	CIT	Scots pine	75	0	-
2	CIT	Scots pine	90	0	-
3	CIT	Scots pine	50	0	-
4	CIT	Scots pine	140	0	-
5	CIT	Scots pine	100	0	-
6	CIT	Scots pine	150	0	-
7	CIT	Scots pine	90	0	-
8	CIT	Scots pine	38	0	-
9	CT	Norway spruce	22	0	-
10	CT	mixed	57	0	-
11	CT	mixed	110	0	-
12	CT	mixed	35	0	-
13	CT	mixed	70	0	-
14	CT	mixed	105	20	-
15	CT	Scots pine	144	1	-
16	CT	Scots pine	141	18	-
17	CT	Scots pine	25	0	-
18	CT	Scots pine	120	0	-
19	VT	Scots pine	43	24	0
20	VT	Scots pine	95	2	24
21	VT	downy birch	16	1	8
22	VT	downy birch	68	0	169
23	VT	downy birch	25	0	132
24	VT	silver birch	70	0	108
25	VT	Scots pine	31	0	4
26	VT	Scots pine	71	0	4
27	VT	Scots pine	107	0	5
28	VT	Scots pine	145	5	5
29	VT	Scots pine	30	0	0
30	VT	Scots pine	100	0	3
31	VT	Scots pine	150	4	0
32	VT	Scots pine	230	0	19
33	VT	Scots pine	27	0	0
34	VT	Scots pine	50	0	14
35	VT	Scots pine	120	0	0
36	VT	Scots pine	160	0	0
37	VT	Scots pine	180	0	0
38	VT	mixed	22	0	19
39	VT	mixed	70	1	7
40	VT	Scots pine	24	0	5
41	VT	mixed	45	0	13
42	VT	Scots pine	45	25	18
43	VT	Scots pine	123	0	23
44	VT	Scots pine	85	0	8
45	VT	Scots pine	100	26	18
46	VT	Scots pine	120	40	7
47	MT	Norway spruce	48	0	14
48	MT	Norway spruce	110	4	17
49	MT	Norway spruce	140	3	22

50	MT	Norway spruce	80	0	0
51	MT	Norway spruce	114	0	0
52	MT	Norway spruce	35	2	5
53	MT	Norway spruce	90	0	0
54	MT	mixed	15	0	6
55	MT	mixed	116	0	49
56	MT	Norway spruce	27	0	10
57	MT	Norway spruce	60	0	4
58	MT	Norway spruce	90	0	31
59	MT	Norway spruce	130	1	7
60	MT	Norway spruce	165	0	0
61	MT	mixed	90	15	34
62	MT	mixed	135	18	0
63	MT	mixed	79	47	22
64	MT	mixed	96	21	55
65	MT	mixed	126	4	0
66	MT	Scots pine	153	7	41
67	MT	mixed	51	6	21
68	MT	mixed	33	3	69
69	MT	Norway spruce	175	8	19
70	MT	mixed	6	0	1
71	MT	Scots pine	112	13	25
72	MT	mixed	133	0	0
73	MT	mixed	15	0	4
74	MT	mixed	73	15	60
75	OMT	mixed	158	17	66
76	OMT	mixed	203	30	89
77	OMT	Norway spruce	48	25	0
78	OMT	Norway spruce	65	10	0
79	OMT	Norway spruce	90	47	0
80	OMT	Norway spruce	60	0	35
81	OMT	mixed	95	0	115
82	OMT	mixed	103	3	47
83	OMT	Norway spruce	141	70	18
84	OMT	mixed	69	0	14
85	OMT	mixed	100	5	107
86	OMT	Norway spruce	48	0	0
87	OMT	mixed	86	6	107
88	OMT	mixed	25	0	27
89	OMT	mixed	20	0	40
90	OMT	Scots pine	127	6	23
91	OaMT	mixed	50	0	18
92	OaMT	mixed	113	4	42
93	OaMT	mixed	40	2	154
94	OaMT	mixed	60	0	107
95	OaMT	mixed	25	0	137
96	OaMT	mixed	60	0	49
97	OaMT	mixed	125	21	33
98	OaMT	mixed	71	0	142
99	OaMT	downy birch	81	0	276
100	OaMT	mixed	35	0	9

Table S1.4 Biodiversity values and carbon values of the sites

Site number	*BD value, current*	*BD value, potential*	*tCO_2, current storage*	*10 €/ tCO_2*	*20 €/ tCO_2*	*50 €/ tCO_2*	*tCO_2, potential sink*	*10 €/ tCO_2*	*20 €/ tCO_2*	*50 €/ tCO_2*
1	0.24	0.29	27	271	542	1356	143	327	654	1635
2	0.26	0.33	134	1336	2673	6682	246	560	1120	2801
3	0.22	0.28	41	414	828	2069	215	491	982	2454
4	0.30	0.30	46	464	928	2321	165	377	753	1883
5	0.27	0.32	106	1058	2116	5291	67	153	307	767
6	0.30	0.31	137	1366	2733	6832	117	268	535	1339
7	0.26	0.31	76	755	1511	3776	124	282	564	1411
8	0.21	0.26	31	310	620	1549	181	413	825	2063
9	0.20	0.30	32	319	638	1594	228	520	1040	2599
10	0.23	0.32	75	750	1499	3748	261	595	1190	2974
11	0.27	0.34	188	1875	3750	9375	273	623	1245	3113
12	0.21	0.38	129	1286	2572	6431	277	632	1265	3161
13	0.24	0.34	127	1274	2549	6372	192	438	876	2189
14	0.36	0.33	153	1526	3053	7632	253	578	1156	2889
15	0.31	0.44	264	2641	5281	13204	292	666	1333	3331
16	0.39	0.33	127	1271	2542	6354	173	396	791	1978
17	0.20	0.27	53	526	1053	2632	153	348	697	1742
18	0.28	0.35	140	1399	2799	6997	132	302	604	1510
19	0.26	0.55	380	3799	7599	18997	331	755	1510	3775
20	0.30	0.69	261	2609	5218	13045	312	713	1426	3564
21	0.18	0.34	8	80	160	401	164	375	749	1873
22	0.33	0.97	171	1712	3424	8560	156	356	713	1782
23	0.27	0.85	133	1325	2651	6627	379	866	1731	4328
24	0.33	0.57	109	1094	2189	5472	228	520	1039	2598
25	0.18	0.58	132	1321	2641	6603	454	1035	2071	5177
26	0.21	0.54	111	1113	2225	5563	527	1203	2406	6015
27	0.24	0.39	178	1779	3559	8897	394	900	1800	4499
28	0.28	0.41	124	1237	2474	6184	394	900	1799	4498
29	0.17	0.31	57	570	1139	2848	292	665	1331	3327
30	0.23	0.46	226	2263	4526	11314	202	461	921	2303
31	0.26	0.33	226	2256	4512	11279	219	500	1001	2501
32	0.30	0.53	411	4109	8219	20546	173	396	791	1978
33	0.17	0.43	137	1372	2745	6862	369	843	1685	4214
34	0.22	0.63	125	1253	2506	6265	328	747	1495	3736
35	0.24	0.42	282	2825	5649	14124	278	633	1266	3165
36	0.24	0.43	424	4240	8480	21200	164	373	746	1865
37	0.24	0.40	377	3775	7550	18875	195	446	891	2229
38	0.20	0.84	107	1071	2143	5357	368	840	1680	4199
39	0.22	0.41	253	2526	5052	12630	229	522	1044	2611
40	0.17	0.57	214	2135	4270	10675	460	1050	2100	5250
41	0.21	0.54	115	1148	2296	5740	282	643	1285	3213
42	0.33	0.88	211	2115	4229	10573	407	929	1858	4645
43	0.31	0.86	515	5148	10297	25741	146	332	665	1661
44	0.23	0.77	375	3752	7505	18762	436	994	1988	4969
45	0.36	0.57	387	3868	7736	19339	117	266	532	1331
46	0.46	0.46	329	3286	6571	16428	142	323	647	1617
47	0.22	0.86	286	2859	5717	14293	615	1403	2807	7017
48	0.31	0.68	180	1796	3591	8978	661	1507	3014	7535
49	0.33	0.59	379	3785	7570	18926	542	1237	2475	6187
50	0.21	0.33	258	2584	5168	12921	312	713	1425	3563
51	0.24	0.34	334	3342	6685	16712	267	610	1219	3048
52	0.19	0.43	162	1616	3233	8082	483	1102	2203	5508

53	0.22	0.29	197	1969	3937	9843	337	768	1537	3842
54	0.17	0.50	42	416	831	2078	466	1064	2128	5319
55	0.39	0.82	315	3148	6297	15742	344	786	1572	3929
56	0.19	0.86	156	1557	3114	7785	361	824	1647	4118
57	0.20	0.48	211	2115	4230	10575	375	856	1712	4280
58	0.31	0.65	363	3633	7265	18163	206	470	941	2352
59	0.27	0.45	282	2821	5642	14105	321	731	1463	3657
60	0.24	0.36	303	3032	6065	15161	267	608	1217	3042
61	0.40	0.81	284	2836	5672	14179	441	1005	2011	5026
62	0.32	0.60	139	1388	2776	6939	475	1084	2167	5418
63	0.50	0.94	495	4952	9904	24760	352	804	1607	4018
64	0.51	1.00	529	5291	10583	26456	329	751	1502	3756
65	0.26	0.34	506	5060	10120	25299	387	882	1763	4408
66	0.42	1.00	529	5290	10579	26448	209	476	952	2379
67	0.26	0.55	129	1289	2577	6443	500	1141	2282	5706
68	0.30	0.70	125	1251	2501	6253	269	614	1228	3070
69	0.35	0.57	301	3008	6016	15040	236	538	1077	2692
70	0.15	0.34	4	36	72	180	473	1078	2156	5390
71	0.39	0.83	388	3876	7753	19382	381	869	1737	4343
72	0.24	0.60	502	5015	10031	25077	382	871	1743	4356
73	0.16	0.82	7	66	132	330	399	909	1819	4547
74	0.43	0.66	314	3140	6279	15698	346	789	1579	3947
75	0.53	0.61	380	3797	7594	18985	286	652	1304	3260
76	0.63	0.93	310	3102	6205	15512	358	816	1632	4079
77	0.27	0.28	289	2887	5775	14437	499	1138	2276	5691
78	0.23	0.33	488	4877	9755	24387	448	1021	2042	5106
79	0.41	0.40	409	4089	8179	20447	419	955	1910	4776
80	0.28	0.58	370	3697	7394	18486	478	1089	2179	5447
81	0.37	0.84	397	3968	7936	19841	421	959	1919	4797
82	0.39	0.66	284	2841	5683	14207	449	53	2050	5124
83	0.69	0.55	196	1962	3924	9811	414	943	1887	4717
84	0.24	0.95	267	2667	5334	13335	431	982	1965	4911
85	0.41	1.00	438	4380	8761	21901	476	1085	2171	5426
86	0.18	0.56	599	5987	11974	29934	363	827	1654	4135
87	0.39	0.94	484	4843	9687	24217	440	1005	2009	5023
88	0.22	0.56	27	271	542	1355	344	784	1568	3919
89	0.25	0.69	40	401	802	2005	275	628	1255	3138
90	0.35	0.50	222	2218	4436	11089	150	343	685	1713
91	0.24	0.61	89	886	1771	4428	533	1215	2430	6074
92	0.40	0.79	363	3633	7267	18166	371	846	1692	4229
93	0.46	0.96	152	1521	3043	7607	361	823	1647	4117
94	0.50	0.74	153	1535	3069	7673	395	900	1800	4500
95	0.42	0.80	138	1376	2751	6878	258	588	1175	2939
96	0.35	0.64	173	1733	3466	8665	372	849	1697	4243
97	0.41	0.56	382	3821	7642	19104	273	622	1244	3109
98	0.53	1.00	433	4334	8668	21669	526	1201	2402	6005
99	0.55	1.00	306	3056	6113	15282	493	1124	2248	5621
100	0.20	0.53	380	3797	7594	18985	515	1174	2348	5869

Appendix S2

Table S2.1 Results with a carbon premium of 10 €/tCO_2 (price-elastic supply)

	**CO_2 current**	**CO_2 potential**
Conservation costs, total, €	444 800	374 003
BD current value × area	0.31 x 58 = 18.2	0.30 x 62 = 18.4
Storage, tCO_2	11 970	10 960
Potential sink, tCO_2	16 680	20 350
Combined index value, current	38.2	36.7
Combined index value, potential	43.5	49.2
Average stand age, years	83	71

Table S2.2 Results with a carbon premium of 30 €/tCO_2 (price-elastic supply)

	**CO_2 current**	**CO_2 potential**
Conservation costs, total, €	675 870	463 730
BD current value × area	0.31 x 62 = 19.4	0.29 x 65 = 19.2
Storage, tCO_2	11 760	11 160
Potential sink, tCO_2	18 420	20 530
Combined index value, current	39.0	37.8
Combined index value, potential	47.2	50.2
Average stand age, years	80	75

Table S2.3 Results with a carbon premium of 40 €/tCO_2 (price-elastic supply)

	**CO_2 current**	**CO_2 potential**
Conservation costs, total, €	790 730	507 790
BD current value × area	0.31 x 64 = 19.9	0.29 x 65 = 19.1
Storage, tCO_2	11 530	10 730
Potential sink, tCO_2	19 490	20 960
Combined index value, current	39.2	37.0
Combined index value, potential	49.4	50.8
Average stand age, years	78	73

Table S2.4 Results with a carbon premium of 50 €/tCO_2 (price-elastic supply)

	**CO_2 current**	**CO_2 potential**
Conservation costs, total, €	905 570	552 720
BD current value × area	0.31 × 66 = 20.4	0.29 × 67 = 19.7
Storage, tCO_2	11 610	11 020
Potential sink, tCO_2	20 050	21 270
Combined index value, current	39.8	38.1
Combined index value, potential	50.7	51.8
Average stand age, years	76	71

Table S2.5 Results under inelastic supply, 20 € tCO_2^{-1}

	**CO_2 current**	**CO_2 potential**
Conservation costs, total, €	560 950	418 860
BD current value × area	0.32 x 57 = 18.1	0.30 x 60 = 18.2
Storage, tCO_2	11 790	11 290
Potential sink, tCO_2	16 250	19 310
Combined index value, current	37.9	37.0
Combined index value, potential	42.8	47.4
Average stand age, years	85	77

Table S2.6 Results when potential biodiversity values are considered instead of current ones, 20 €

	**Baseline**	**CO_2 current**	**CO_2 potential**
Conservation costs, total, €	299 980	560 060	418 850
BD current value × area	0.31 × 56 = 17.2	0.31 × 62 = 19.0	0.30 × 62 = 18.6
Storage, tCO_2	10 670	11 510	10 960
Potential sink, tCO_2	17 510	19 550	20 500
Combined index value, current	35.1	38.2	36.9
Combined index value, potential	43.7	48.6	49.6
Average stand age, years	77	75	71
